# Prognostic value of imaging-based ATN profiles in a memory clinic cohort

**DOI:** 10.1007/s00259-023-06311-3

**Published:** 2023-06-26

**Authors:** Débora E. Peretti, Federica Ribaldi, Max Scheffler, Christian Chicherio, Giovanni B. Frisoni, Valentina Garibotto

**Affiliations:** 1 Laboratory of Neuroimaging and Innovative Molecular Tracers (NIMTlab), Geneva University Neurocentre and Faculty of Medicine, University of Geneva, Geneva, Switzerland; 2 Laboratory of Neuroimaging of Aging (LANVIE), University of Geneva, Geneva, Switzerland; 3 Geneva Memory Centre, Department of Rehabilitation and Geriatrics, Geneva University Hospitals, Geneva, Switzerland; 4 Division of Radiology, Geneva University Hospitals, Geneva, Switzerland; 5 Centre for Interdisciplinary Study of Gerontology and Vulnerability (CIGEV), University of Geneva, Geneva, Switzerland; 6 Division of Nuclear Medicine and Molecular Imaging, Geneva University Hospitals, Geneva, Switzerland; 7 Centre for Biomedical Imaging, University of Geneva, Geneva, Switzerland

**Keywords:** ATN profile, Cognitive decline, Alzheimer’s disease, Positron emission tomography

## Abstract

**Purpose:**

The ATN model represents a research framework used to classify subjects based on the presence or absence of Alzheimer’s disease (AD) pathology through biomarkers for amyloid (A), tau (T), and neurodegeneration (N). The aim of this study was to assess the relationship between ATN profiles defined through imaging and cognitive decline in a memory clinic cohort.

**Methods:**

One hundred-eight patients from the memory clinic of Geneva University Hospitals underwent complete clinical and neuropsychological evaluation at baseline and 23 ± 5 months after inclusion, magnetic resonance imaging, amyloid and tau PET scans. ATN profiles were divided into four groups: normal, AD pathological change (AD-PC: A + T-N-, A + T-N +), AD pathology (AD-P: A + T + N-, A + T + N +), and suspected non-AD pathology (SNAP: A-T + N-, A-T-N + , A-T + N +).

**Results:**

Mini-Mental State Examination (MMSE) scores were significantly different among groups, both at baseline and follow-up, with the normal group having higher average MMSE scores than the other groups. MMSE scores changed significantly after 2 years only in AD-PC and AD-P groups. AD-P profile classification also had the largest number of decliners at follow-up (55%) and the steepest global cognitive decline compared to the normal group. Cox regression showed that participants within the AD-P group had a higher risk of cognitive decline (HR = 6.15, CI = 2.59–14.59), followed by AD-PC (HR = 3.16, CI = 1.17–8.52).

**Conclusion:**

Of the different group classifications, AD-P was found to have the most significant effect on cognitive decline over a period of 2 years, highlighting the value of both amyloid and tau PET molecular imaging as prognostic imaging biomarkers in clinical practice.

**Supplementary information:**

The online version contains supplementary material available at 10.1007/s00259-023-06311-3.

## Introduction

The current research framework for the classification of subjects within the Alzheimer’s disease (AD) spectrum is based on the presence or absence of biomarker pathology: extracellular amyloid plaques (A), neurofibrillary tau tangles (T), and neurodegeneration (N) [1]. Each biomarker is considered independent from each other, and subjects can be classified as positive or negative for each of them. Together, the three biomarkers compose the ATN model, where each subject is classified with an unbiased descriptive pathological biomarker profile. Despite being considered independent from each other, a dynamic model for a pathological cascade has been proposed, where the disease starts with A deposits, followed by T, which finally leads to N [2]. Therefore, a temporal evolution of biomarker status is expected with the progression to AD. Indeed, previous studies have shown that the ATN classification is correlated with a clinical progression towards dementia [3, 4], memory worsening [5] and cognitive decline [6–10]. These ATN profiles can be categorised into four larger groups: normal biomarkers, AD pathological change (AD-PC; profiles with partial characteristic AD pathology, i.e., positive for A but not for T pathology), AD pathology (AD-P; profiles with characteristic AD pathology, i.e., positive for A and T pathology), and suspected non-Alzheimer’s disease pathology (SNAP; profiles negative for A but positive for T and/or N).

Positron emission tomography (PET) and magnetic resonance imaging (MRI) are imaging modalities that have been shown to provide good markers for individually predicting disease progression when compared to fluid biomarkers [4, 8, 11, 12]. PET images can be used to show in vivo deposits of A and T, while MRI provides a measure of brain atrophy, which is related to N. The ensuing measurements can be quantified, and subjects can be classified into different ATN profiles based on either visual inspection of the images or specific quantitative thresholds.

As imaging includes expensive techniques to assess biomarkers, other methods for measuring the same biomarkers have been used. However, PET and MRI have been shown to be the most precise approaches to predict disease progression [8]. Furthermore, previous studies have focused on cohorts of patients situated at an early stage of the AD spectrum and in research cohorts. A longitudinal study investigating the effect of ATN profiles, measured only through imaging techniques, in global cognitive decline has not been performed yet. Furthermore, a study of this design using data from subjects with a variety of diagnoses and cognitive statuses at baseline, evaluated at a memory clinic level, is also at need. Therefore, the aim of this study was to assess the relationship between ATN profiles and cognitive decline, measured through a decline in MMSE scores, in a memory clinic population.

## Materials and methods

### Subjects

A cohort of 108 subjects was selected from an ongoing study at the Geneva Memory Clinic at the Geneva University Hospitals (HUG), Geneva, Switzerland. These subjects were selected from a larger cohort of available subjects since they fitted the inclusion criteria: (1) amyloid and tau PET imaging performed within 12 months of each other (average 3.8 ± 5.1 months), (2) 3D T1 MRI scans performed within a year from the PET image (average 0.4 ± 10.5 months), (3) neuropsychological assessment at baseline was performed within 6 months of PET imaging (average 0.4 ± 5.8 months), (4) a follow-up neuropsychological assessment was performed after an average of 24 months from baseline (average 23.0 ± 11.1 months). The local review board (Cantonal Research Ethics Commission, Geneva, Switzerland) approved the study, which has been conducted in concordance with the principles of the Declaration of Helsinki and the International Conference on Harmonisation Good Clinical Practice. All subjects provided written informed consent to have collected data be used in research.

This memory clinic cohort included subjects with a variety of diagnoses at baseline: healthy control individuals (HC), and patients with subjective cognitive decline (SCD), mild cognitive impairment (MCI), or dementia. Diagnosis was based on a clinical assessment combined with the results of the neuropsychological assessment. Subjects with SCD were evaluated at the Geneva Memory Clinic with a self-experience of deterioration in cognitive abilities but did not present objective cognitive impairment through formal neuropsychological testing [13]. MCI patients presented objective cognitive impairment and no functional impairing in everyday life [14]. Individuals were diagnosed with dementia if they matched MCI requirement but differ from MCI subjects for the impairment in everyday life [15]. Dementia patients were further diagnosed based on probable aetiology: two suspected non-AD dementia, one dementia due to stroke, one Parkinson’s dementia, and 11 AD dementia. Diagnosis was used to characterise the included population, but it was not included as a significant variable in the assessment of cognitive decline of each profile. Neuropsychological assessment also included a Mini-Mental State Examination (MMSE), which was used in this study as a measure of global cognition and to assess cognitive decline [16]. Cognitive decline was defined by a decrease of one MMSE point per year [17]. A subset of the cohort underwent other neuropsychological tests for verbal episodic memory (free and cued selective remining test — RLRI16 in French), attention (trail making test — TMT), and verbal fluency (categorical and phonemic).

### Imaging acquisition and processing

MRI exams were performed at the Division of Radiology Department of HUG. 3D T1 images were acquired using a 3 Tesla Siemens Magnetom Skyra scanner (Siemens Healthineers, Erlangen, Germany) equipped with a 64-channel head coil and were acquired in close accordance with IMI pharmacog WP5/European ADNI sequences and published procedures [18]. In summary, a field of view of 256 mm, 0.9–1 mm slice thickness, 1819–1930 ms repetition time, 2.19–2.4 ms echo time, 8° flip angle, and no fat suppression were used.

PET imaging was performed at the Nuclear Medicine and Molecular Imaging Division of the HUG. For amyloid imaging, 42 subjects were injected with 204 ± 23 MBq of [^18^F]florbetapir, and images were acquired 50 min after intravenous administration of the radiotracer (3 × 5 min image frames that were averaged into a single image). The remaining 66 subjects were scanned using 171 ± 19 MBq of [^18^F]flutemetamol, and images were acquired 90 min after intravenous administration of the radiotracer (4 × 5 min image frames that were averaged into a single image). For tau images, subjects were injected with 200.70 ± 18.98 MBq of [^18^F]flortaucipir, and images were acquired 75 min after tracer injection (6 × 5 min image frames that were averaged into a single image). All images were acquired using a Siemens Biograph PET scanner (Siemens Medical Solutions, USA), reconstructed using a 3D OSEM algorithm (4 iterations, 8 subsets), a 2 mm Gaussian convolution kernel, corrected for dead time, normalisation, attenuation, and sensitivity. All commercially available radiotracers were synthesised at radiopharmaceutical Good Manufacturing Practice laboratories.

All images were processed at the Geneva Memory Centre of HUG, Geneva, Switzerland, using SPM12 (Wellcome Trust Centre for Neuroimaging, London, UK) and MATLAB R2018b version 9.5 (MathWorks Inc., Sherborn, USA). Firstly, 3D T1 MRI images were aligned to the anterior commissure. Then, they were normalised to the Montreal Neurologic Institute (MNI) space using tissue probability maps [19]. PET images were aligned to the subject’s respective MRI images and then, using the MRI transformation matrix, they were transformed into the MNI space.

Cortical reconstruction and volumetric segmentation of T1 images were performed using Freesurfer (v7, recon-all [20]). Right and left hippocampal volumes were extracted, averaged, and normalised to the total intracranial volume. Amyloid PET data were converted into the centiloid scale [21], so that data from the different radiotracers could be equally compared, and global centiloid values were used in this study. For the tau PET images, standardised uptake value rations (SUVR) were generated using the cerebellar crus as a reference tissue [22, 23], and data were extracted using the automated anatomic labelling atlas 3 [24, 25]. Regional data were combined in a weighted average according to the Simplified Temporal-Occipital Classification (STOC) into four main regions: medial temporal lobe (MTL; hippocampus, amygdala, parahippocampus, and fusiform gyrus), lateral temporal lobe (LTL; Heschl, temporal inferior and middle gyrus), superior temporal gyrus (STG), and the primary visual cortex (PVC) [23]. Furthermore, a global tau SUVR value was calculated based on a combination of the amygdala, parahippocampus, middle occipital gyrus, and inferior temporal gyrus [26] for further analyses. As a supplementary analysis, N was also estimated using the cortical thickness of an AD-specific region of interest (weighted average cortical thickness in the entorhinal, inferior temporal, middle temporal, and fusiform) [27], setting a threshold of 2.57 for discriminating between N + and N − subjects [28].

### ATN classification

Positivity for each biomarker was defined based on previously published thresholds. Subjects were considered A + when the centiloid value was above 12 [29]. Tau status was assessed based on the STOC model [23]. Each region from the STOC classification, described on the previous section (MTL, LTL, STG, and PVC) was considered positive if its SUVR value was above 1.28, and tau STOC stage was defined based on these positivises. Stages 0 (all negative) and 1 (MTL positive only) were considered T-, while stages 2 (LTL or MTL + LTL positive) 3 (stage 2 + STG), and 4 (stage 3 + PVC) were considered T + . Neurodegeneration positivity was decided based on each subject’s ratio of hippocampal volume to total intracranial volume, a variable called from here on as ‘hippocampal ratio’. If this value was below 0.00215, subjects were considered N + [27]. Combining biomarker positivity information, subjects were then classified into ATN profiles. Due to the small number of subjects in some of the ATN classifications, profiles were then combined into larger groups that comprise of more than one profile (with the exception of one classification): normal (A-T-N-), AD-PC (A + T-N-, A + T-N +), AD-P (A + T + N-, A + T + N +), and SNAP (A-T + N-, A-T-N + , A-T + N +).

### Statistical analysis

Kruskal-Wallis test and Dunn tests for multiple corrections using false discovery rate (FDR) were performed to explore differences in age, years of education, MMSE (both at baseline and follow up), centiloid, global tau SUVR, and hippocampal ratio between groups. Significant differences between baseline and follow-up MMSE scores were assessed using paired Wilcoxon tests for each group individually.

Linear mixed models were used to estimate the association between ATN profiles at baseline and changes in MMSE scores in a 2-year period considering the normal group as the reference. Cox proportional hazards analysis was performed to evaluate the association between each group (normal biomarker group as reference) and cognitive decline, defined as a decrease of one MMSE point per year [16]. Linear mixed models were also used for the association between A, T, and N biomarkers individually at baseline and changes in MMSE scores at follow-up using negative biomarkers as reference, and cognitive decline, using the same criterium.

Neuropsychological tests (RLRI16, TMT, and verbal fluency) were analysed using the same methods as for the MMSE: a Kruskal-Wallis test and Dunn test for multiple correction using FDG to compare between ATN groups, and a paired Wilcoxon test to compare between baseline and follow up scores.

A *p*-value of 0.05 was considered the significance threshold for all analyses, which were performed using RStudio (version 2022.07.1, R version 4.2.1). For the Kruskal-Wallis and Dunn tests, the *FSA* R package (version 0.9.3) was used. For the linear mixed models, the *lme4* package (version 1.1-29) was used. The *survival* package (version 3.4-0) was used for the Cox proportional hazards estimations and the *survminer* package (version 0.4.9) was used for plotting Kaplan-Meyer curves.

## Results

### Population

**Fig. 1:**
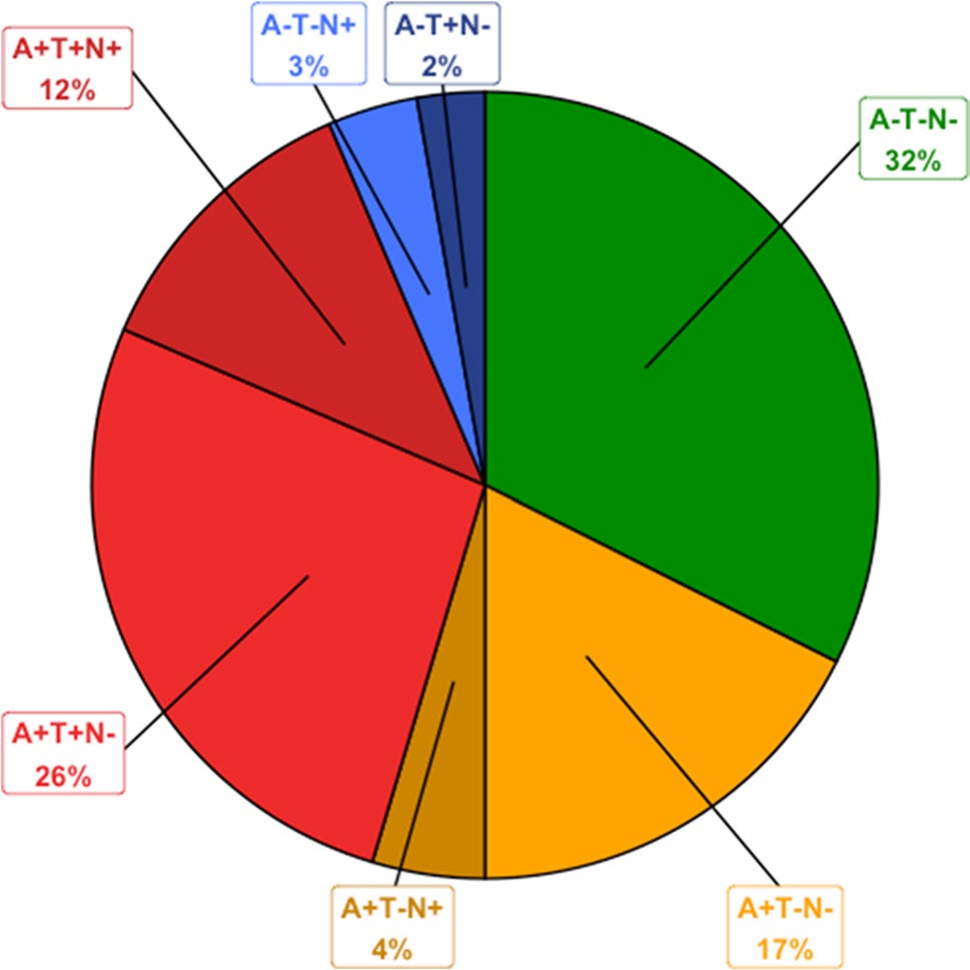
Pie chart illustrating the distribution of ATN profiles and their percentage. In green, the normal group (negative for all biomarkers); in shades of orange, the profiles included in the AD-PC (A + T −) group; in shades of red, the profiles included in the AD-P (A + T +) group; and in blue, the profiles included in the SNAP group

Figure 1 shows the distribution of ATN profiles and groups. Table 1 shows the demographic, clinical, cognitive, and imaging characteristics of each ATN group at baseline. Education was not significantly different between the groups. Age was significantly different between normal and AD-PC groups, with the latter having a significantly higher age in comparison to the former. MMSE was significantly higher in the normal group when compared to the AD-P and SNAP groups, while in the AD-PC group it was significantly higher than in the AD-P group. Centiloid values were significantly higher in the AD-PC and AD-P groups when compared to normal and SNAP subjects, but not between each other. Global tau SUVR was higher in the AD-P group when compared to all others. Finally, hippocampal ratio was significantly higher in the normal individuals when compared to all other groups. A total of 4 subjects were included as HC, 26 were diagnosed as SCD, 63 as MCI, and 15 as Dementia at baseline. The characteristics of each group are summarised in Supplementary Table 1. No significant differences in population demographics were found when using cortical thickness to define N (Supplementary Table 2).

**Table 1:** Demographic, cognitive, and imaging characteristics of subjects included in the study by ATN groups. Reported *p*-values resulted from Kruskal-Wallis ANOVA. Dunn tests were used for post hoc analysis with Benjamin-Hochberg correction for multiple comparisons. Superscript letters indicate groups showing significant differences at post-hoc comparisons: a > b, c > d

	Normal (*n* = 35)	AD-PC (*n* = 24)	AD-P (*n* = 42)	SNAP (*n* = 7)	*p*-value
Included profiles	A-T-N-	A+T-N- A+T-N +	A+T+N- A+T+N+	A-T+N- A-T-N+ A-T+N+	-
Age (y)	70 ± 7^b^	76 ± 7^a^	74 ± 6	74 ± 6	< 0.01
Gender (M/F)	17/18	17/7	19/23	1/6	-
Education (y)	15 ± 4	14 ± 4	13 ± 5	13 ± 4	0.35
Diagnosis (HC/SCD/MCI/Dementia)	2/18/14/1	2/4/15/3	0/2/31/9	0/2/3/2	-
MMSE	28 ± 2^a^	27 ± 3^c^	24 ± 5^b,d^	25 ± 6^b^	< 0.01
Centiloid	-5 ± 9^b,d^	58 ± 42^c^	88 ± 29^a^	-8 ± 8^b,d^	< 0.01
Global Tau SUVR	1.12 ± 0.09^b^	1.16 ± 0.10^b^	1.61 ± 0.25^a^	1.22 ± 0.16^b^	< 0.01
Hippocampal Ratio (× 10^−3^)	2.5 ± 0.1^a^	2.3 ± 0.3^b^	2.3 ± 0.2^b^	2.2 ± 0.3^b^	< 0.01

*HC*, healthy controls; *SCD*, subjective cognitive decline; *MCI*, mild cognitive impairment; *MMSE*, mini-mental state examination; *A*, amyloid; *T*, tau; *N*, neurodegeneration; *n*, number of subjects; *AD*, Alzheimer’s disease; *AD-PC*, AD pathologic change; *AD-P*, AD pathology; *SNAP*, suspected non-Alzheimer’s disease pathology

### Cognitive follow-up

**Fig. 2:**
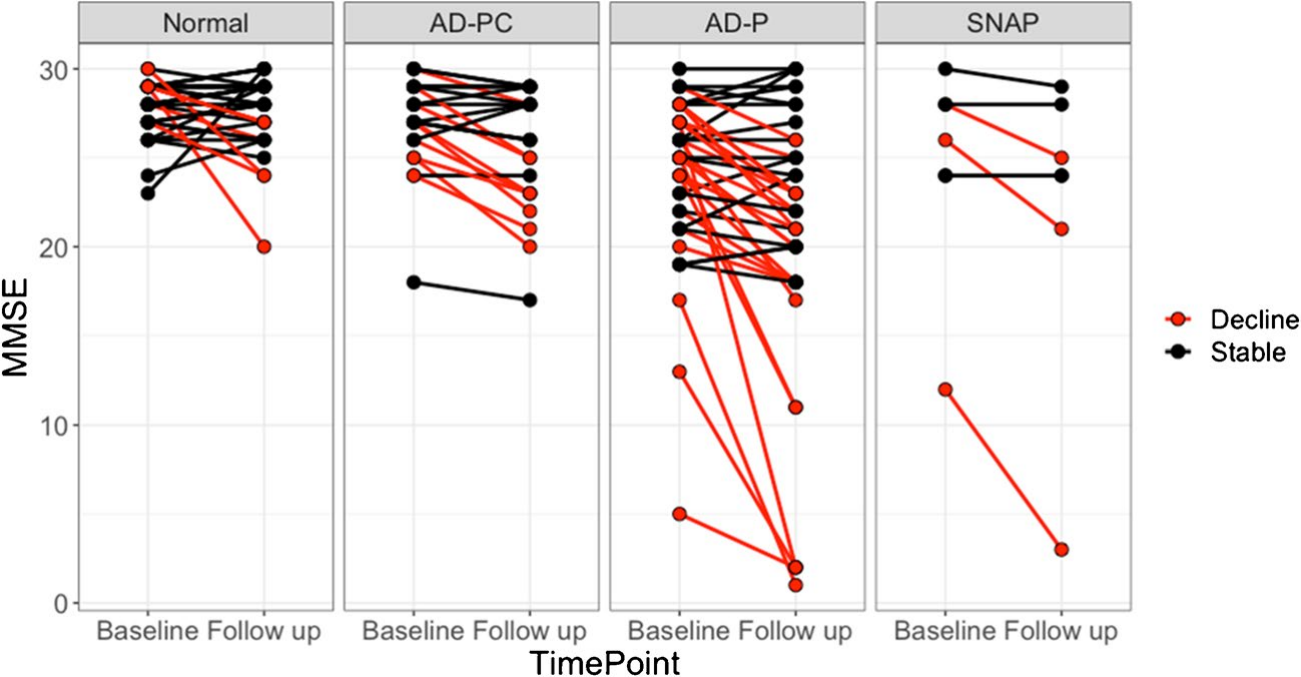
Individual MMSE scores per subject by ATN group. Lines connect points that belong to the same subject, expressing evolutionary trajectory. Each column represents a different ATN group. Red colour represents subjects that significantly declined, and black colour, stable subjects

Table 2 shows MMSE scores per group at baseline and follow-up. Scores were significantly different between groups at both time points, with the normal group having higher MMSE values in comparison to AD-PC and SNAP groups, and the AD-PC group also having higher MMSE values when compared to the AD-P group. However, scores were significantly different between time points in the AD-PC and AD-P groups alone. Furthermore, the AD-P group had the largest percentage of decliners (55%). Figure 2 presents a spaghetti plot of MMSE changes between baseline and follow-up by group, with red lines denoting subjects with significant decline.

**Table 2:** Average and standard deviation of MMSE scores by ATN group at baseline and follow-up, and percentage of subjects with significant cognitive decline by profile. Fourth row contains *p*-values from paired Wilcoxon tests between baseline and follow-up MMSE scores by group. Fifth row shows numbers of subjects that declined at follow-up and the percentage of decliners for each profile. Last column displays *p*-values from the Kruskal-Wallis ANOVA comparing MMSE scores by time points. Superscript letters indicate groups showing significant differences at post hoc comparisons using Dunn tests with Benjamin-Hochberg correction for multiple comparisons: a > b, c > d

ATN Groups	Normal (*n* = 35)	AD-PC (*n* = 24)	AD-P (*n* = 42)	SNAP (*n* = 7)	*p*-value
Baseline	28 ± 2^a^	27 ± 3^c^	24 ± 5^b,d^	25 ± 6^b^	< 0.01
Follow-up	28 ± 2^a^	26 ± 3^c^	21 ± 8^b,d^	22 ± 9^b^	< 0.01
*p*-value	0.55	< 0.01	< 0.01	0.10	-
n Decliners (%)	7 (20)	9 (38)	23 (55)	3 (43)	-
Cox model	1 (reference)	3.16 [1.17–8.52]	6.15 [2.59–14.59]	n.s	-

Abbreviations: *A*, amyloid; *T*, tau; *N*, neurodegeneration; *n*, number of subjects; *n.s.*, not significant; *AD*, Alzheimer’s disease; *AD-PC*, AD pathological change; *AD-P*, AD pathology; *SNAP*, suspected non-AD pathology

For the memory (RLRI16), attention (TMT), and verbal fluency (categorical fruits) tests, no significant differences between groups were found at baseline, but groups were significantly different at follow-up. However, only the AD-P group showed differences in scores between baseline and follow-up in the sum of free recalls, sum of total recalls and delayed total recall, TMTA tests. The phonemic verbal fluency test showed no significant differences between groups neither at baseline nor at follow-up. Furthermore, no significant differences between baseline and follow-up were found in each of the groups. Results for the neuropsychological assessments of the specific domains are presented in Supplementary Table 3 and Supplementary Fig. 1.

### ATN prediction of cognitive decline

A linear mixed model was used to estimate the association between groups and global cognitive status measured through MMSE scores corrected for the effect of time. Table 3 shows estimated baseline MMSE scores and change in 2 years by ATN group. Significant associations were found only in the AD-P group (β =  − 3.81, *p* < 0.01). Furthermore, the same group had significant interactions with time when measuring cognitive decline through MMSE scores, showing a consistent effect (β =  − 3.21, *p* < 0.01). Correcting the model for age, gender, and education did not significantly change the results.

**Table 3:** Linear mixed model results assessing the relationship between each ATN profile at baseline and at follow-up with cognitive decline measured through MMSE scores standardised to the A-T-N- profile. The model was fitted with random intercepts and slopes. *p*-values are the result of the interaction between profiles and time points. The β estimate at baseline represent the adjusted regression coefficient showing the association between groups and baseline MMSE results with the normal group as the reference. The β estimate at follow-up shows the adjusted regression coefficient showing the association between ATN group with annual decline in MMSE score, in comparison to the normal group as the reference

ATN Groups	Baseline			Follow-up		
	β	Confidence Interval	*p*-value	β	Confidence Interval	*p*-value
AD-PC	− 0.82	− 3.28–1.65	0.52	− 1.13	− 3.21–0.95	0.29
AD-P	− 3.81	− 5.94–-1.68	< 0.01	− 3.21	− 5.01– − 1.42	< 0.01
SNAP	− 3.29	− 7.14–0.57	0.09	− 2.29	− 5.54–0.97	0.17

Abbreviations: *A*, amyloid; *T*, tau; *N*, neurodegeneration; *AD*, Alzheimer’s disease; *AD-PC*, AD pathological change; *AD-P*, AD pathology; *β baseline*, adjusted regression coefficient showing the association between profile classification and baseline MMSE result with the normal group as the reference; *β follow-up*, regression coefficient showing the association between ATN group with annual decline in MMSE score in comparison to the normal group as the reference

Supplementary Table 4 shows the results of the linear mixed models used to estimate the association between A, T, and N biomarkers separately and cognitive decline measure through MMSE scores corrected for the effect of time. Significant associations were found for the T biomarker at follow-up (β =  − 1.96, *p* = 0.04). Correcting the model for age, gender, and education did not significantly change the results.

Supplementary Table 5 shows the results of the linear mixed model used to estimate the association between groups and global cognitive status measured though MMSE scores corrected for the effect of time with N measured through cortical thickness. As for N measured through hippocampal ratio, significant association were only found in the AD-P group (β =  − 3.22, *p* < 0.01). Furthermore, the same group had significant interactions with time when measuring cognitive decline through MMSE scores, showing a consistent effect (β =  − 3.07, *p* < 0.01).

### ATN profile risk of cognitive decline

**Fig. 3:**
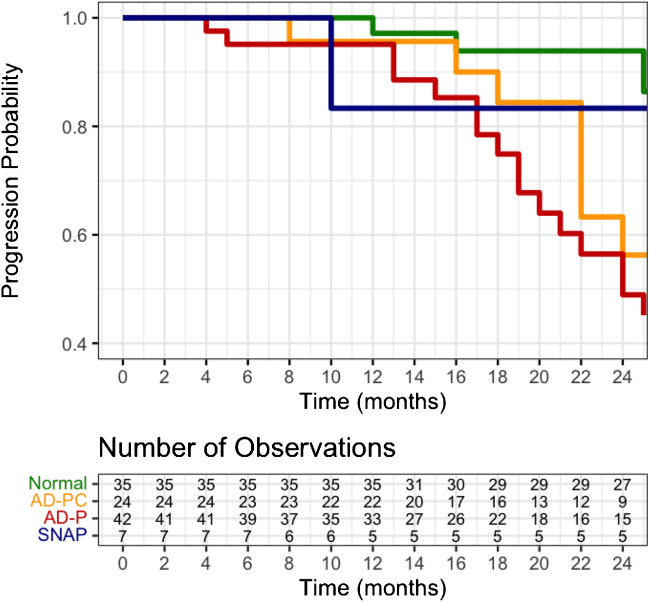
Kaplan-Meyer curves illustrate cognitive decline defined as an MMSE loss of 1 point per year. Separate lines represent each of the ATN groups. Numbers at risk at every 2-month time point are depicted in the risk table below the graph

Table 2 shows the complete results from the Cox proportional hazard analysis. This analysis showed that compared to the normal group, subjects in the AD-PC and AD-P groups were at increased risk of cognitive decline with an incremental increase in hazard ration (HR) (HR 3.16 [1.17–8.52], and HR 6.15 [2.59–14.59], respectively). The SNAP group did not show a significant increase in risk of cognitive decline. Figure 3 shows the Kaplan-Meyer curves illustrating cognitive decline per ATN group.

## Discussion

The aim of this study was to assess the prognostic value of different ATN groups in cognitive decline in a memory clinic population. To this end, subjects with PET and MRI scans, and two independent cognitive assessments conducted on average 2 years apart were selected from a prospective cohort. While 33% of the included subjects presented a normal biomarker profile (A-T-N-), 22% of the subjects presented an A + T- profile and were classified as being within the AD-PC group, and 39% with an A + T + profile which were considered within the AD-P group. The use of ATN stratification has the potential to offer complementary knowledge to clinical and genetic information to predict cognitive decline. Different profiles are expected to progress differently over time because of additive effects of accumulated pathological proteins. Moreover, it is expected that the more pronounced the underlying pathological process is, the faster a patient will decline over time. This study showed that positivity for an AD biomarker in itself is sufficient to predict short-time cognitive decline. Independently of classification, all groups showed an average MMSE score that decreased at follow-up, except for the normal group. However, only A + individuals within the AD-PC and AD-P groups, had significantly different MMSE scores at follow-up when compared to baseline (Table 2).

Most of AD drug trials are focused on amyloid interventions, and include amyloid status information alone. Indeed, it was observed that subjects within the AD continuum presented significant short-time changes in MMSE scores, in comparison to baseline measures (Table 2), supporting the hypothesis that amyloid positivity is a risk factor for cognitive decline. However, there is a significant interplay between different biomarkers that affects disease progression. AD-P subjects had not only MMSE scores significantly lower than AD-PC at baseline and follow-up but also a larger number of subjects who progressed within a 2-year time frame, highlighting the added value of T in assessing cognitive impairment and cognitive decline. Although amyloid positivity is a risk factor for AD and is correlated to disease progression, there is a significant number of A + subjects that do not decline [30]. Furthermore, studies have shown that other factors might help in avoiding cognitive decline even in the presence of amyloid and tau pathologies, such as cognitive reserve [31], diet [32], education [33], lifestyle [31, 34], and others [35, 36]. The identification of patients’ ATN profiles could be used to assess possible therapeutic interventions as profiles decline cognitively at different rates. The urgency of treatment can also be assessed based on the profile, as patients positive for amyloid and tau deposition tend to progress at a faster rate than compared to negative profiles.

AD progression and AD patients’ cognitive decline has been shown to be largely influenced by positivity for the ε4 allele of the apolipoprotein E gene (APOE4) [37]. However, not all subjects of the cohort used in this study had information regarding the presence of the APOE4 allele. Nonetheless, previous studies on the decline of subjects stratified into ATN profiles have shown that short-time progression is independent of APOE4 presence [5, 10, 11]. Therefore, although the inclusion of APOE4 status might improve model prediction, it is unlikely that it would have significantly changed the results found in this study.

Differently from previous results, the only profiles able to predict global cognitive decline in this study were those of the AD-PC and AD-P groups. Previous studies have found that subjects without amyloidosis but with tau pathology significantly declined in a 2-year follow-up period [3]. This study could not confirm this finding in this cohort possibly due to the small sample of subjects in the SNAP group, but also related to the different approaches to assess cognitive decline. Few published works measured cognitive decline using MMSE scores, whereas different domains of cognition individually instead of global cognition were more frequently assessed. Furthermore, while 2 years is a long enough time to state short-time cognitive decline, most studies focused on longer follow-up periods. In addition, it is important to notice that the large variance in the estimation in the linear mixed models applied in this study could also be related to the smaller samples of subjects in some of the groups.

An interesting point to further explore regarding cognitive decline in ATN groups could be the changes of individual biomarker profiles over time. However, most of the subjects included in this study did not undergo follow-up PET and MRI scans. Further studies focusing on the temporal evolution of ATN positivity are still needed. Moreover, imaging techniques also offer the option of staging disease progression [38–41], which would provide different subclassifications within each profile using non-dichotomised A, T, and N biomarker values. Regional uptake in AD-specific regions could provide a better prediction of disease progression. For A, frontal, parietal and occipital regions have been found to be of relevance for identifying specific amyloid deposits [42]. However, their applicability to a standardised general measure as the centiloid scale and the comparability across different amyloid radiotracers should be specifically tested. For T, uptake in regions related to Braak stages [38], or larger and more easily atlas-defined regions such as the medial temporal lobe, lateral temporal lobe, superior temporal gyrus, and the primary visual cortex as defined by the STOC model [23] could be suggested. Finally, N can be expressed as a measure not only of relative hippocampal volume [27], but also hippocampal cortical thickness [43] or through some automated score measurement of AD pattern expression in FDG PET scans [44, 45].

Neurodegeneration is considered, in the ATN framework, as a non-specific marker for AD, and it can be defined not only through MRI, as in this study, but also through [18F]Fluorodeoxyglucose (FDG) [1]. Using a different approach to estimate N might lead to different profile classifications due to the fundamental differences between techniques [46]. In this study, the definition of the N threshold for positivity classification was done based on the ratio of the hippocampal volume relative to the total intracranial volume: a measure of hippocampal atrophy. In comparison, FDG PET assessment for N classification is based in a wider assessment of brain regions that present a hypometabolic pattern [47–49]. Although these measurements are correlated [50], they do not measure the same aspect of neurodegeneration and, therefore, different results of profile classifications and a higher sensitivity could be expected using FDG as a biomarker for N instead of MRI [51]. Finally, due to the non-specific nature of N as a biomarker in the ATN framework, the classification of profile subgroups was mostly based on A and T positivity, while N was mostly used to differentiate SNAP patients from normal subjects.

The use of an imaging approach to assess ATN groups, the approach that has been shown to be better correlated to disease progression [8], is the strongest point of this study. In addition to this, the use of a population recruited directly from a memory clinic results in findings that are implementable in clinical practice, in contrary to research populations. Although this study was not focused on subjects’ clinical status, it is interesting to notice that the SCD group was mostly characterised with a normal profile, which is in line with a previous publication that focused on SCD individuals alone [3]. Meanwhile, MCI patients were mostly classified as AD-PC or AD-P. The definition of MCI was solely based on cognitive complaints and did not consider the underlying cause for such complaints. However, as the ATN research framework was created with the purpose of classifying AD, and AD is the most prevalent form of dementia [52], it was expected for MCI population to be classified mostly as AD-PC or AD-P.

Neuroimaging modalities provide reliable biomarkers for the classification of ATN profiles and to assess longitudinal cognitive decline. However, imaging can be an expensive and burdensome technique for the patients. Cerebral spinal fluid is more commonly used in clinical practice, and also provides markers for ATN classification that are related to cognitive decline [7, 53, 54]. Furthermore, in the recent years, a great development of techniques for the assessment of plasma-based biomarkers has been achieved. These markers would be even less invasive for patients as they could easily be implemented in routine blood examens. Studies assessing ATN classification and the prognostic value of this technique to assess ATN biomarkers have shown promising results [10, 55]. Yet, the measurement of plasma-based biomarker levels is still under development and requires validation in different settings for it to be available in clinical routine [56]. Finally, it is important to point out that only neuroimaging techniques provide a visualisation of pathology deposition that allow for disease staging and regional uptake assessment that, as previously discussed, could provide a more precise evaluation of individual prognosis.

This study has a number of limitations worth discussing. Firstly, the population included in this study was a memory clinic population and although the majority of the patients that are referred to the clinic are within the AD spectrum, not all subjects included in this study were classified as dementia due to AD but included other diagnoses. However, given the lower prevalence of non-AD diagnoses, our study did not allow to stratify the prognostic analysis by diagnosis. Secondly, the classification of subjects in the different subcategories of the ATN framework led to small subgroups that were then pooled into larger categories (AD-PC, AD-P, and SNAP subjects), preventing firm conclusions on each specific combination. The ATN framework includes specific markers for AD pathology and a non-specific marker of neurodegeneration (N). Properly investigated, N markers (MRI and FDG PET when available) could also provide specific information on non-AD degenerative disturbances, such as investigating frontal abnormalities. However, given the lower prevalence of these conditions, this study did not use markers for N other than targeting AD and, thus, cannot conclude on their prognostic value in this cohort. Furthermore, a 2-year follow-up period was used, which is not a sensitive measure for slow and gradual decline, namely in healthy individuals, and only allows to identify subjects at a higher risk of declining. Finally, only a minority of the included subjects had a complete neuropsychological evaluation at both time points and for this reason mainly the MMSE as a global measure of cognition was used to measure decline. In particular, the small number of SNAP and AD-PC subjects might have limited the ability of this study to detect significant changes between these groups.

## Conclusion

The stratification of subjects into ATN groups allows for an accurate estimate of the risk of cognitive decline. In our cohort, the AD-P group had the largest change in MMSE scores and at the highest risk of significant cognitive decline over a short follow-up period.

### Supplementary Information

Below is the link to the electronic supplementary material.

Supplementary file1 (DOCX 232 KB)

## Data Availability

Datasets generated and/or analyzed during the current study are available with the corresponding author upon reasonable request.
