# Supplementary material for: Prognostic value of imaging-based ATN profiles in a memory clinic cohort
Source: Eur J Nucl Med Mol Imaging. 2023 Jun 26;50(11):3313–23. doi: 10.1007/s00259-023-06311-3 (PMC10542279; doi:10.1007/s00259-023-06311-3)
Supplement: Supplementary file 1 — Supplementary file1 (DOCX 232 KB) [file 259_2023_6311_MOESM1_ESM.docx]

**Supplementary Table 1.** Demographic, cognitive, and imaging characteristics of subjects included in the study by diagnosis. Reported p-values results from Kruskal-Wallis ANOVA. Dunn tests were used for post-hoc analysis with Benjamin-Hochberg correction for multiple comparisons. Superscript letters indicate groups showing significant differences at post-hoc comparisons: a > b, c > d, e > f. Dementia diagnosis included two suspected non-Alzheimer’s dementia patients, one stroke patient, one Parkinson’s disease dementia patient, and 11 Alzheimer’s Dementia patients.

|  | **HC**  **(n = 4)** | **SCD**  **(n = 26)** | **MCI**  **(n = 63)** | **Dementia**  **(n = 15)** | ***p*-value** |
| --- | --- | --- | --- | --- | --- |
| **Age (y)** | 72 ± 5 | 72 ± 7 | 73 ± 7 | 74 ± 7 | 0.72 |
| **Gender (M/F)** | 0/4 | 10/16 | 36/27 | 8/7 | - |
| **Education (y)** | 13 ± 4 | 17 ± 5^a^ | 14 ± 4^b^ | 11 ± 4^b^ | < 0.01 |
| **Classification**  **(Normal/AD-PC/**  **AD-P/SNAP)** | 2/2/0/0 | 18/4/2/2 | 14/15/31/3 | 1/3/9/2 | - |
| **MMSE** | 29 ± 1^a^ | 28 ± 1^c^ | 26 ± 3^b,d,e^ | 20 ± 6^b,d,f^ | < 0.01 |
| **Centiloid** | 35 ± 57 | 10 ± 32^b,d^ | 57 ± 50^c^ | 60 ± 47^a^ | < 0.01 |
| **Global Tau SUVR** | 1.07 ± 0.12^b,d^ | 1.14 ± 0.14^b,d^ | 1.37 ± 0.28^a^ | 1.54 ± 0.35^c^ | < 0.01 |
| **Hippocampal Ratio**  **(x10^-3^)** | 2.4 ± 0.1 | 2.5 ± 0.2^a^ | 2.3 ± 0.3^b^ | 2.2 ± 0.3^b^ | < 0.01 |

HC = healthy control, SCD = subjective cognitive decline, MCI = mild cognitive impairment, MMSE = mini-mental state examination, n = number of subjects, AD-PC = AD pathologic change, AD-P = AD pathology, SNAP = suspected non-Alzheimer’s disease pathology.

**Supplementary Table 2.** Demographic, cognitive, and imaging characteristics of subjects included in the study by ATN groups (with N measured through cortical thickness). Reported *p*-values resulted from Kruskal-Wallis ANOVA. Dunn tests were used for post-hoc analysis with Benjamin-Hochberg correction for multiple comparisons. Superscript letters indicate groups showing significant differences at post-hoc comparisons: a > b, c > d

|  | **Normal**  **(n = 37)** | **AD-PC**  **(n = 24)** | **AD-P**  **(n = 42)** | **SNAP**  **(n = 5)** | ***p*-value** |
| --- | --- | --- | --- | --- | --- |
| **Included Profiles** | A-T-N- | A+T-N-  A+T-N+ | A+T+N-  A+T+N+ | A-T+N-  A-T-N+  A-T+N+ | - |
| **Age (y)** | 70 ± 6^b^ | 76 ± 7^a^ | 74 ± 6 | 71 ± 11 | 0.01 |
| **Gender (M/F)** | 21/16 | 17/7 | 19/23 | 3/2 | - |
| **Education (y)** | 15 ± 4 | 14 ± 4 | 13 ± 5 | 13 ± 2 | 0.39 |
| **Diagnosis**  **(HC/SCD/MCI/Dementia)** | 2/18/14/3 | 2/4/15/3 | 0/2/31/9 | 0/2/3/0 | - |
| **MMSE** | 27 ± 3^a^ | 27 ± 3^c^ | 24 ± 5^b,d^ | 28 ± 2 | < 0.01 |
| **Centiloid** | -5 ± 9^b,d^ | 58 ± 42^c^ | 88 ± 29^a^ | -8 ± 9^b,d^ | < 0.01 |
| **Global Tau SUVR** | 1.12 ± 0.09^b^ | 1.16 ± 0.10^b^ | 1.61 ± 0.25^a^ | 1.22 ± 0.19^b^ | < 0.01 |
| **Composite Cortical Thickness** | 2.8 ± 0.1^a^ | 2.7 ± 0.2 | 2.6 ± 0.2^b^ | 2.7 ± 0.1 | < 0.01 |

HC = healthy controls, SCD = subjective cognitive decline, MCI = mild cognitive impairment, MMSE = mini-mental state examination, A = amyloid, T = tau, N = neurodegeneration, n = number of subjects, AD = Alzheimer’s disease, AD-PC = AD pathologic change, AD-P = AD pathology, SNAP = suspected non-Alzheimer’s disease pathology

**Supplementary Table 3.** Average and standard deviation of neuropsychological tests by ATN group at baseline and follow up. Rows containing “*p*-value Wilcoxon” contain the *p*-values from paired Wilcoxon tests comparing baseline and follow up scores for each test. Last column displays *p*-values from the Kruskal-Wallis ANOVA comparing group scores by time point. Superscript letters indicate groups showing significant differences at post-hog comparisons using Dunn tests with Benjamin-Hochberg correction for multiple comparisons: a > b

| **ATN Groups** | **Normal** | **AD-PC** | **AD-P** | **SNAP** | ***p*-value** |
| --- | --- | --- | --- | --- | --- |
| **RLRI16 Sum of free recalls** | n = 23 | n = 7 | n = 10 | n = 3 | - |
| **Baseline** | 27.5 ± 6.9 | 26.0 ± 7.7 | 21.7 ± 7.9 | 23 ± 5.2 | 0.24 |
| **Follow up** | 27.4 ± 7.3^a^ | 25.7 ± 7.9 | 14.1 ± 10.3^b^ | 18.3 ± 8.6 | < 0.01 |
| ***p*-value Wilcoxon** | 0.83 | 0.83 | 0.02 | 0.37 | - |
| **RLRI16 Sum of total recalls** | n = 23 | n = 7 | n = 10 | n = 3 | - |
| **Baseline** | 44.4 ± 4.3 | 43.6 ± 4.2 | 40.9 ± 7.5 | 44.7 ± 5.8 | 0.54 |
| **Follow up** | 44.9 ± 3.5^a^ | 43.4 ± 5.5 | 30.8 ± 12.7^b^ | 34.3 ± 13.3 | < 0.01 |
| ***p*-value Wilcoxon** | 0.38 | 0.89 | 0.03 | 0.25 | - |
| **RLRI16 Delayed free recall** | n = 23 | n = 8 | n = 9 | n = 3 | - |
| **Baseline** | 11.3 ± 3.5 | 9.6 ± 4.4 | 7.8 ± 3.5 | 9.3 ± 1.2 | 0.07 |
| **Follow up** | 11.1 ± 2.8 | 10.6 ± 4.1 | 6.3 ± 5.1 | 5.3 ± 5.0 | 0.04 |
| ***p*-value Wilcoxon** | 0.42 | 0.23 | 0.18 | 0.37 | - |
| **RLRI16 Delayed total recall** | n = 23 | n = 8 | n = 9 | n = 3 | - |
| **Baseline** | 15.4 ± 1.2 | 14.5 ± 1.8 | 13.2 ± 3.3 | 15.3 ± 1.2 | 0.17 |
| **Follow up** | 15.6 ± 0.7^a^ | 14.5 ± 2.8^a^ | 7.1 ± 6.2^b^ | 10.7 ± 9.2 | < 0.01 |
| ***p*-value Wilcoxon** | 0.43 | 1.0 | 0.03 | 1.0 | - |
| **TMT A** | n = 26 | n = 9 | n = 15 | n = 3 | - |
| **Baseline** | 40.3 ± 13.2 | 42.4 ± 14.5 | 46.3 ± 9.6 | 47.0 ± 17.3 | 0.43 |
| **Follow up** | 43.8 ± 17.8^a^ | 41.8 ± 11.9^a^ | 60.2 ± 14.0^b^ | 49.3 ± 14.7 | < 0.01 |
| ***p*-value Wilcoxon** | 0.46 | 1.0 | < 0.01 | 1.0 | - |
| **TMT B** | n = 26 | n = 9 | n = 11 | n = 3 | - |
| **Baseline** | 92.8 ± 45.7 | 106.0 ± 40.8 | 96.9 ± 24.7 | 134.0 ± 26.9 | 0.13 |
| **Follow up** | 91.8 ± 33.9^b^ | 125.0 ± 51.1 | 129.0 ± 46.5^a^ | 98.0 ± 12.5 | 0.04 |
| ***p*-value Wilcoxon** | 0.31 | 0.25 | 0.05 | 0.25 | - |
| **TMT B-A** | n = 26 | n = 9 | n = 11 | n = 3 | - |
| **Baseline** | 52.5 ± 38.2 | 63.8 ± 34.3 | 50.2 ± 25.0 | 87.3 ± 10.5 | 0.16 |
| **Follow up** | 48.0 ± 32.1 | 82.9 ± 49.3 | 70.4 ± 41.4 | 48.7 ± 8.3 | 0.04 |
| ***p*-value Wilcoxon** | 0.93 | 0.16 | 0.15 | 0.25 | - |
| **Verbal Fluency: Categorical Fruits** | n = 19 | n = 9 | n = 18 | n = 4 | - |
| **Baseline** | 18.7 ± 5.8^a^ | 18.6 ± 8.0 | 14.3 ± 4.6^b^ | 15.2 ± 2.2 | 0.05 |
| **Follow up** | 17.8 ± 5.8^a^ | 17.8 ± 4.5 | 11.1 ± 6.3^b^ | 13.0 ± 4.83 | 0.01 |
| ***p*-value Wilcoxon** | 0.38 | 0.72 | 0.03 | 0.69 | - |
| **Verbal Fluency: Phonemic** | n = 20 | n = 9 | n = 15 | n = 4 | - |
| **Baseline** | 16.8 ± 5.6 | 18.3 ± 7.0 | 17.1 ± 5.6 | 13.5 ± 5.6 | 0.64 |
| **Follow up** | 19.1 ± 7.8 | 19.8 ± 6.3 | 12.7 ± 8.8 | 13.5 ± 8.5 | 0.21 |
| ***p*-value Wilcoxon** | 0.06 | 0.50 | 0.03 | 1.0 | - |

Abbreviations: A = amyloid, T = tau, N = neurodegeneration, n = number of subjects, AD = Alzheimer’s disease, AD-PC = AD pathological change, AD-P = AD pathology, SNAP = suspected non-AD pathology, RLRI16 = free and cued selective reminding test, TMT = trail making test

**Supplementary Table 4.** Linear mixed model results assessing the relationship between A, T, and N biomarkers at baseline and at follow-up with cognitive decline measured through MMSE scores standardised to being negative to these biomarkers (A-, T-, N-). The model was fitted with random intercepts and slopes. *p*-values are the result of the interaction between profiles and time points. The β estimate at baseline represent the adjusted regression coefficient showing the association between groups and baseline MMSE results with the negative biomarkers as reference. The β estimate at follow-up shows the adjusted regression coefficient showing the association between each biomarker individually with annual decline in MMSE score, in comparison to negative biomarkers as reference.

| **Biomarker** | **Baseline** | | | **Follow up** | | |
| --- | --- | --- | --- | --- | --- | --- |
|  | **β** | **Confidence Interval** | ***p*-value** | **β** | **Confidence Interval** | ***p*-value** |
| **A+** | -0.41 | -2.65 – 1.83 | 0.72 | -0.90 | -2.80 – 1.00 | 0.35 |
| **T+** | -2.22 | -4.42 – -0.02 | 0.04 | -1.96 | -3.82 – -0.09 | 0.04 |
| **N+** | -2.87 | -5.15 – -0.58 | 0.01 | -0.39 | -2.33 – 1.55 | 0.69 |

Abbreviations: A = amyloid, T = tau, N = neurodegeneration, β baseline = adjusted regression coefficient showing the association between biomarkers and baseline MMSE result with the negative biomarkers as reference, β follow-up = regression coefficient showing the association between biomarkers with annual decline in MMSE score in comparison to negative biomarkers as the reference.

**Supplementary Table 5.** Linear mixed model results assessing the relationship between each ATN profile at baseline and at follow-up with cognitive decline measured through MMSE scores standardised to the A-T-N- profile (with N status measured through cortical thickness). The model was fitted with random intercepts and slopes. *p*-values are the result of the interaction between profiles and time points. The β estimate at baseline represent the adjusted regression coefficient showing the association between groups and baseline MMSE results with the normal group as the reference. The β estimate at follow-up shows the adjusted regression coefficient showing the association between ATN group with annual decline in MMSE score, in comparison to the normal group as the reference.

| **ATN Groups** | **Baseline** | | | **Follow up** | | |
| --- | --- | --- | --- | --- | --- | --- |
|  | **β** | **Confidence Interval** | ***p*-value** | **β** | **Confidence Interval** | ***p*-value** |
| **AD-PC** | -0.23 | -2.73 – 2.27 | 0.86 | -0.98 | -3.05 – 1.08 | 0.35 |
| **AD-P** | -3.22 | -5.37 – -1.07 | < 0.01 | -3.07 | -4.85 – -1.29 | < 0.01 |
| **SNAP** | 0.33 | -4.22 – 4.88 | 0.89 | -1.97 | -5.73 – 1.79 | 0.30 |

Abbreviations: A = amyloid, T = tau, N = neurodegeneration, AD = Alzheimer’s disease, AD-PC = AD pathological change, AD-P = AD pathology, β baseline = adjusted regression coefficient showing the association between profile classification and baseline MMSE result with the normal group as the reference, β follow-up = regression coefficient showing the association between ATN group with annual decline in MMSE score in comparison to the normal group as the reference.

**Supplementary Fig 1.** Individual scores for neuropsychological tests by ATN group. Lines connect points that belong to the same subject, expressing evolutionary trajectory. Each column represents a different ATN group, and each row a different neuropsychological test.
